# Interaction of C/EBP-beta and NF-Y factors constrains activity levels of the nutritionally controlled promoter IA expressing the acetyl-CoA carboxylase-alpha gene in cattle

**DOI:** 10.1186/1471-2199-13-21

**Published:** 2012-06-27

**Authors:** Xuanming Shi, Cornelia C Metges, Hans-Martin Seyfert

**Affiliations:** 1 Research Unit for Molecular Biology, Leibniz Institute for Farm Animal Biology (FBN), Wilhelm-Stahl-Allee 2, D-18196, Dummerstorf, Germany; 2 Research Unit for Nutritional Physiology, Leibniz Institute for Farm Animal Biology (FBN), Wilhelm-Stahl-Allee 2, D-18196, Dummerstorf, Germany; 3 Department of Physiology and Developmental Biology, UT Southwestern Medical Center, 5323 Harry Hines Blvd., Dallas, TX, 75390-9133, USA

**Keywords:** ACC-alpha, Bos taurus, CCAAT-enhancer binding protein, Fat synthesis, Gene regulation, Nuclear factor Y

## Abstract

**Background:**

The enzyme acetyl-CoA carboxylase-alpha (ACC-α) is rate limiting for de novo fatty acid synthesis. Among the four promoters expressing the bovine gene, promoter IA (PIA) is dominantly active in lipogenic tissues. This promoter is in principal repressed but activated under favorable nutritional conditions. Previous analyses already coarsely delineated the repressive elements on the distal promoter but did not resolve the molecular nature of the repressor. Knowledge about the molecular functioning of this repressor is fundamental to understanding the nutrition mediated regulation of PIA activity. We analyzed here the molecular mechanism calibrating PIA activity.

**Results:**

We finely mapped the repressor binding sites in reporter gene assays and demonstrate together with Electrophoretic Mobility Shift Assays that nuclear factor-Y (NF-Y) and CCAAT/enhancer binding protein-β (C/EBPβ) each separately repress PIA activity by binding to their cognate low affinity sites, located on distal elements of the promoter. Simultaneous binding of both factors results in strongest repression. Paradoxically, over expression of NFY factors, but also - and even more so - of C/EBPβ significantly activated the promoter when bound to high affinity sites on the proximal promoter. However, co-transfection experiments revealed that NF-Y may eventually diminish the strong stimulatory effect of C/EBPβ at the proximal PIA in a dose dependent fashion. We validated by chromatin immunoprecipitation, that NF-Y and C/EBP factors may physically interact.

**Conclusion:**

The proximal promoter segment of PIA appears to be principally in an active state, since even minute concentrations of both, NF-Y and C/EBPβ factors can saturate the high affinity activator sites. Higher factor concentrations will saturate the low affinity repressive sites on the distal promoter resulting in reduced and calibrated promoter activity. Based on measurements of the mRNA concentrations of those factors in different tissues we propose that the interplay of both factors may set tissue-specific limits for PIA activity.

## Background

Acetyl-CoA carboxylase-alpha (ACC-α) is the rate-limiting enzyme in de novo synthesis of long-chain fatty acids [1]. It thus plays an important role in controlling lipid metabolism [2]. Dysregulation of lipid metabolism in human and mouse is known to result in metabolic diseases, such as obesity and diabetes, and may also accompany cancer [3,4]. It may also result in severe metabolic disorders in lactating cows [5]. Hence, an improved understanding of the regulation of ACC-α activity might help developing new strategies to avoid health problems associated with perturbed fat metabolism in keeping of dairy cattle.

Multiple promoters express the mammalian ACC-α encoding-gene. Their activities are differentially influenced by diets and hormones (e.g. insulin and glucagon) [1,6]. The bovine gene was previously known to be expressed by three promoters [7-9]. Among them, the 5´- most located promoter was designated as “PI” and was supposed to be nutritionally regulated. However, we recently identified an additional fourth promoter located ~41 kbp more upstream, similar as previously reported from sheep [10]. We validated in separate experiments that this promoter is also active in cattle, mainly in the brain (EMBL Acc No FN185962, FN185963). To avoid confusion regarding the designation of the various promoters, we adopt here the recently proposed nomenclature of ACC-α promoters (Additional file 1: Figure S1) [6]. Thus, we now name, for cattle the most 5´-located promoter as PI and designate the nutritionally regulated promoter as PIA (previously known as “PI” [7]). We keep the previous designations for the constitutive promoter PII [8] and the mammary gland expressed and lactationally activated PIII [9].

PIA (Additional file 1: Figure S1), but not the other promoters, is known as the dominant ACC-α promoter of lipogenic tissues, such as liver, adipose tissues and mammary gland in ruminant and rodent species [11-13]. Activity of this promoter is controlled by insulin through an element binding the upstream stimulatory factors(USF1 or USF2) [14]. Glucose regulates PIA via a carbohydrate response element (ChoRE) located on the proximal promoter [15].

Promoter PIA is basically repressed [7,16,17]. Coarse mapping of structural elements necessary for repression of PIA in cattle had revealed that the repressor consists of potentially three distinct elements which are separated by more than 1000 bp [7]. The molecular nature of the repressor and the repressive mechanism controlling PIA activity remained unknown, so far. Cooperation of at least two of these structural elements was shown to be necessary in order to exert repression [7]. PIA from rat is also repressed by a distal element [16]. Over- expression of C/EBPα or − β factors may overcome repression of the rat PIA by binding to the proximal promoter and stimulate its activity in reporter gene assays [16,17].

C/EBP factors are ubiquitously expressed and are known to play an essential role in controlling the expression of genes with relevance for energy metabolism [18,19]. The C/EBP factor family includes six structurally related members (α, β, δ, ϵ, γ, ζ). Their specific N-termini encompass not only activation domains but also repression domains. The conserved C-termini constitute a DNA binding domain while an internal leucine zipper domain is necessary for factor dimerization [20]. These factors have a quite complex repertoire of mechanisms to eventually regulate expression of their target genes, involving factor dimerization, either as homo- or heteromer. Differential phosphorylation and synthesis of different isoforms were also shown to regulate activity of those factors [20,21].

C/EBPα is known to interact with NF-Y factors [22,23]. The ubiquitously expressed NF-Y factor family consists of three subunits, NF-YA, -B and -C. Subunits -B and -C are constitutively expressed, while the expression of NF-YA is regulated [24,25]. NF-YB and -C have to form a dimer before NF-YA can bind to the complex. Only that trimer of NF-Y factors can bind to a CCAAT-box on the DNA. Mutation of three amino acids in the binding domain of the NF-YA subunit abrogates DNA-binding of the entire NF-Y complex [26]. NF-Y may act as a bifunctional regulator by either repressing or activating transcription during different physiological conditions [27].

It was found in rat and goat that the presence of this distal repressor reduces the insulin mediated stimulation of PIA activity [14,15]. Hence, quite obviously does the activity of the repressor influence and eventually bias the analysis of the nutritionally and hormone mediated fine tuning of PIA activity. Therefore, we set out to characterize the molecular nature of that general repressor of PIA in cattle. We first finely mapped the cis-relevant repressive elements and determined in mutation analyses and EMSA assays that NF-Y and C/EBP factors act as repressors. Surprisingly, we found that these factors both are also crucial enhancers of PIA activity, if they are bound to the proximal promoter. We provide evidence that interaction in trans allows the distally bound repressors to inhibit the stimulatory effect of the proximally bound partner factors. Measurements of the endogenous mRNA levels suggest that the proportion of these factors may set coarse tissue-specific limits for the overall PIA promoter activity.

## Results

### PIA is the nutritionally regulated promoter

**Figure 1:**
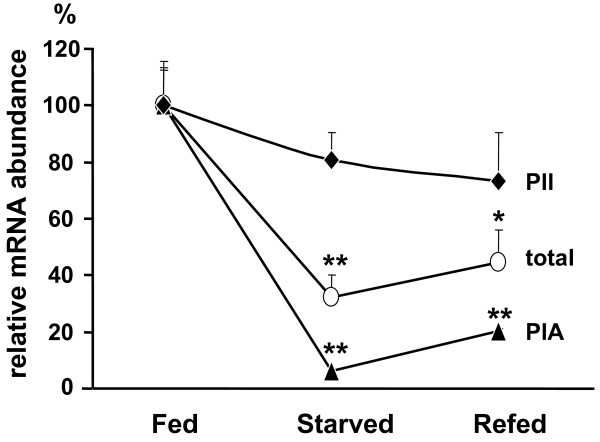
**Effect of starvation and refeeding on the relative abundance of ACC-αmRNA molecules derived from PIA, PII, and from all promoters (total) in the liver.** The relative concentration of mRNA molecules derived from PIA, PII and all ACC-α encoding messages were measured from liver biopsies. These were taken from normally fed animals (Fed), probed again after starvation (43 h, Starved) and after subsequently refeeding them for 29 h (Refed). Values were normalized relative to the mean concentrations as measured from the well fed animals, set as 100 %. Shown are the mean values (±S.E.M., n = 4). Statistical significance of differences was calculated against respective Fed samples (* *P* < 0.05; ** *P* < 0.01).

We validated first that indeed the activity of PIA is nutritionally controlled, using the nutritionally modulated expression of the ACC-α in liver as paradigm. To this end we selected four first lactating cows and took biopsies from their livers. Subsequently the cows were starved for 43 h, and the livers were biopsied again. Sampling was repeated subsequent to refeeding the animals for 29 h again. Starvation decreased the total ACC-α mRNA content down to ~32% of that found in the livers of the well fed animals (Figure 1). This decrease was caused primarily by a cessation of PIA activity, as indicated by a >94% reduction of PIA derived transcripts. The transcripts derived from the house keeping promoter (PII), on the other hand, only decreased by ~19%. The data show that the level of PIA derived transcripts in the liver reflects the metabolic state of the animal, better than the concentration of all ACC-α-encoding messages. Also, gross alterations in PIA activity influence the total amount of ACC-α transcripts. Strong nutrition-dependent regulation of PIA activity in the liver was also recorded in a different experiment, in which a group of six cows was kept on a minimal energy, straw based diet for 60 h [28]. This diet supplied only 16 % of the energy contained in the normal diet having been fed to the control group. The concentration of PIA derived ACC-α transcripts was proportionally reduced, down to 19 % (Table1).

**Table 1:** Relative mRNA levels of ACC-α PIA, NF-YA and C/EBPβ in various tissues under different nutritional conditions

	**Copies**	**Liver**		**Adipose**		**Mammary gland**	
**Diet**		**Straw**	**Normal**	**Fat**	**Starch**	**Starch**	**Fat**
ACC-α PIA	x 10^4^	0.10 ± 0.01 ^a^	0.52 ± 0.22 ^b^	6.81 ± 2.24	8.49 ± 4.86	19.79 ± 4.79	42.85 ± 12.98
NF-YA	x 10^3^	8.94 ± 1.05	7.64 ± 0.71	10.41 ± 0.97	18.57 ± 7.61	8.38 ± 1.48	7.56 ± 1.06
C/EBPβ	x 10^6^	4.37 ± 0.25	3.71 ± 0.80	2.45 ± 0.35	3.08 ± 1.10	0.62 ± 0.09^a^	0.36 ± 0.06 ^b^
NFYA:C/EBPβ	x 10^-3^	2.06 ± 0.24	2.44 ± 0.64	4.69 ± 0.71	6.45 ± 0.90	14.07 ± 1.87^a^	25.89 ± 5.08 ^b^
NEL	MJ	18.0 ± 1.1	110.2 ± 11.2	nd	nd	nd	nd
Fat	g/kg DM	nd	nd	51	28	28	51
Carbohydrate	g/kg DM	nd	nd	31	100	100	31
n	6	6	8	8	8	8	8

Mean values (±S.E.M.). Different superscripts (“a” and “b”) indicate statistically different values (P < 0.05) within the respective tissue. NEL, Net Energy content for Lactation; nd, not determined; n, number of animal.

### Distal NF-Y and C/EBP binding sites are involved in PIA repression

**Figure 2:**
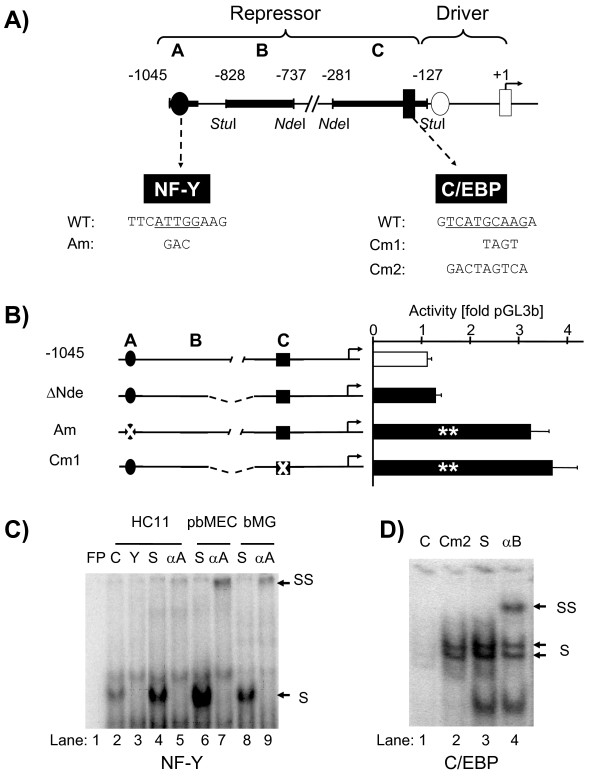
**NF-Y and C/EBP binding sites in element A and C respectively in the distal region are involved in PIA repression.** (**A**) Genomic organization of PIA. The distal region includes three putative repressive elements, as revealed by coarse mapping analyses involving *Stu*I and *Nde*I deletions, respectively (filled boxes, element A, B, C). Element A harbors a NF-Y attachment site (filled circle), and element C contains a C/EBP binding site (filled square). The core binding sequences of those factors and mutational derivatives are shown. The proximal driving region features a NF-Y site (open circle) and a C/EBP binding site (open square). ‘+1’ represents the tsp. (**B**) Abrogation of NF-Y binding and C/EBP binding in the distal PIA relieves repression in reporter gene assay. Shown are mean values (+ S.E.M.) of at least three independent experiments, each assayed in triplicate. Significance of difference relative to construct −1045 (WT) is indicated (** *P <* 0.01). (**C**) EMSA analysis of transcription factors binding to element A. The labeled probe A_WT (Additional file 1: Table S4) is bound by NF-Y factors from nuclear extracts of HC-11, pbMEC and bMG (“S”) and is supershifted (SS) by the addition of antibodies against NF-YA (αA). Lanes: FP, free probe; C and Y, competition with 100-fold molar excess of the unlabeled A_WT probe and a probe harboring an NF-Y consensus site respectively (Additional file 1: Table S4). (**D**) EMSA validation that C/EBP factors bind to element C. Nuclear extracts from HC-11 cells were incubated with the radioactively labeled probe C_WT (Additional file 1: Table S4). Lane: C, competition with 100-fold molar excess of unlabeled C_WT; S, shift; αB, supershift (“SS”) with anti-C/EBP antibodies; Cm2, competition with 100-fold molar excess of the unlabeled probe mutated as shown in Figure 2A.

**Figure 3:**
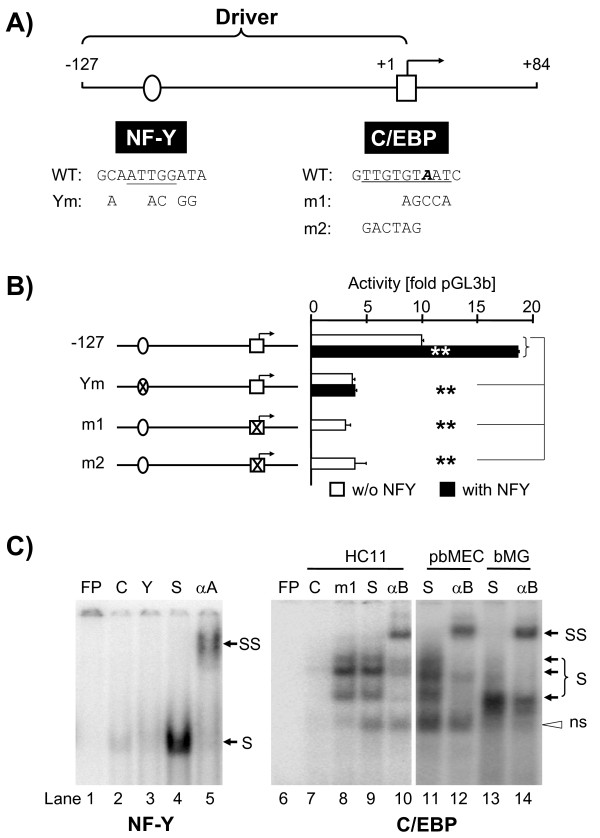
**Binding of NF-Y and C/EBP factors to the proximal PIA drives activity.** (**A**) Map and sequences of the NF-Y and C/EBP binding sites on the proximal PIA. Core motifs (underlined) of the respective binding sequences and mutations are shown, as they have been included in EMSA and reporter gene analyses. The tsp in the C/EBP motif (“A” nucleotide, bold, italicized) is indicated. (**B**) Mutation of the binding sites of NF-Y (Ym) as well as of C/EBP (m1, m2) significantly reduce the reporter genes activity (open columns). Joint co-transfection of expression constructs for all three NF-Y factors (NF-YA -B, -C) significantly increase activity of WT-promoter, but not of the construct Ym. Mean values (+ S.E.M.) from one out of three experiments (Ym) or six independent experiments (C/EBP mutation), always assayed in triplicate and normalized against the WT construct. (**C**) EMSA analyses of the proximal NF-Y and C/EBP binding sites. Left: Nuclear extracts from HEK293 cells over-expressing jointly the factors NF-YA, -B, -C were incubated with the probe harboring the proximal NF-Y binding site (pNF_WT, Additional file 1: Table S4). The shifted band (S) was supershifted (SS) by anti-NF-YA antibodies (αA). Right: The labeled probe was the proximal C/EBP site (pCE_WT, Additional file 1: Table S4). Multiple specific shift bands (S) appeared with nuclear extracts from HC-11 cells, pbMEC, or bMG. Anti-C/EBP antibodies (αB) caused supershifts (SS). Lane designations are similar to Figure 2C and D; m1, competition with 100-fold molar excess of unlabeled probe, mutated as shown in Figure 3A. “ns” represents a non-specific band.

We finely mapped with serial deletions of promoter segments the structural elements contributing to PIA repression in murine mammary epithelial model cells (MEC) HC-11. MEC cells are a relevant model for fatty acid synthesis, since they synthesize in vivo abundant amounts of milk fat [29] and the previous functional characterization of PIA had in part been conducted using these cells [7]. We found that two elements (A and C) from among three potentially relevant elements must be present to repress the PIA activity (Figure 2A and Additional file 1: Figure S2A). Deletion of either element increased the promoter activity > 3-fold. Strongest promoter activation was achieved, if both repressive sites were deleted. We cloned to this end a short segment comprising only 127 bp of the proximal PIA promoter (Driver, Figure 2A and Additional file 1: Figure S2A). This element very actively expresses the reporter gene, resulting in approximately 10-fold of the reporter activity measured from the promoter-less pGL3b (Figure 3B), confirming previous observations [7]. The extent of enhanced activity of this short minimal promoter indicates an additive effect of both repressive sites.

More detailed deletion analyses revealed that the 5´-located half of element A is the key component of the repressive element (Figure 2A and Additional file 1: Figure S2B). Abolishing the NF-Y factor binding site in this element via point mutation increased promoter activity ~3-fold, indicating some relieveof repression (Figure 2B, clone Am). EMSA assays demonstrated that NF-Y factors can indeed bind to a probe harboring the 5´-half of element A (Figure 2C, lane4). We used for these assays nuclear extracts from murine HC-11 cells, from primary bovine MEC cells (pbMEC) or from the lactating udder of a cow (bMG). The specific complex (NF-Y) could be inhibited by the addition of a 100-fold molar excess of a competitor, harboring a NF-Y consensus motif (Figure 2C, lane3) embedded into otherwise completely PIA-unrelated DNA sequence. A rabbit antibody specific for NF-YA supershifted the protein-DNA complex (Figure 2C, lane7, 9). The NF-Y specificity of this supershift is clearly demonstrated, since an antibody directed against C/EBP did not supershift this band (Additional file 1: Figure S2C, lane4). Introducing a mutation (“Am”) into the unlabeled “cold” competitor in EMSA assays abolished competition (Additional file 1: Figure S2C, lane9). The data together suggest that NF-Y factors binding to this site constitute the active component of the 5´-half of the PIA repressor.

We characterized the repressive principle in element C using a similar strategy. Deletion analyses in reporter gene experiments indicated a putative C/EBP binding site as a candidate sequence motif (Figure 2A, B and Additional file 1: Figure S2D). Using this candidate area as a probe in EMSA analyses showed that mutation of the C/EBP binding site abolished factor binding (Figure 2D, lane2, Cm2) and an antibody against C/EBPβ supershifted this band (Figure 2D, lane4). Mutation of the C/EBP-core recognition motif relieved repression to the same extent as deleting that element (compare Figure 2B, clone Cm1 vs Additional file 1: Figure S2A, clone ΔStu). Hence, the relevant DNA-sequence element within the repressive element C is a C/EBP binding site.

Our data together show that the structure of the bovine PIA repressor consists of a distal NF-Y binding site and a more proximally residing C/EBP binding site (Figure 2A). These sites cooperate functionally to exert full repression.

### C/EBP and NF-Y factors activate PIA by binding to proximal promoter segments

Unexpectedly, cloning and co-transfecting together expression constructs for all three different NF-Y factors (A, B, C) consistently increased the activity of our long PIA reporter construct. Moreover, we previously reported that cotransfecting expression constructs for any of the C/EBP-factors -α, -β, -δ, -ϵ increased significantly the activity of this promoter. C/EBPβ had the strongest activation potential [11]. Sequence analysis of the PIA DNA sequence revealed putative binding sites for both factors, indeed, located on the proximal promoter (Figure 3A). These are located on our minimal-127 bp promoter construct. When expression constructs for all three NF-Y subunits (A, B, C) were jointly co-transfected together with the reporter gene, they increased the reporter activity by 60–100% (Figure 3B). Abrogation of the NF-Y binding site via point mutation reduced the promoter activity by ~60% (Figure 3B, clone Ym). In support, co-transfected NF-Y expression constructs did not enhance the activity of the reporter harboring the mutated NF-Y binding site (Figure 3B, clone Ym, black column). EMSA analysis confirmed that over-expressed NF-Y-factors indeed may bind to this element (Figure 3C, lane4). The unlabeled probe bearing a NF-Y consensus binding site (Y) competed away the shift band (Figure 3C, lane3), and anti-NF-YA antibodies (αA) supershifted this band (Figure 3C, lane5).

EMSA assays demonstrated that nuclear extracts from HC-11 clearly shifted a radioactively labeled probe bearing the proximal C/EBP motif (Figure 3C, lane9) which could also be supershifted by antibodies specific for C/EBPβ (Figure 3C, lane10, 12, 14). Similarly, nuclear extracts derived either from pbMEC or bMG also formed several complexes with that probe, some of which could be supershifted by those antibodies. Abrogating this site via point mutation reduced the promoter activity down to ~30% of the WT activity (Figure 3B, clones m1 and m2). Considering that this C/EBP binding site comprises the most 3´-located transcriptional start point (tsp) of PIA [7], we inserted two different mutations into this sequence element, one eliminating the “A” nucleotide forming the WT tsp while the other maintained the tsp (Figure 3B; clones m1 and m2, respectively). Both mutations resulted in a comparable and diminished activity of the reporter. The data together show that C/EBP as well as NF-Y factors activate the proximal PIA promoter.

### The distal sites bind the respective factors with much lower affinities than the proximal sites

**Figure 4:**
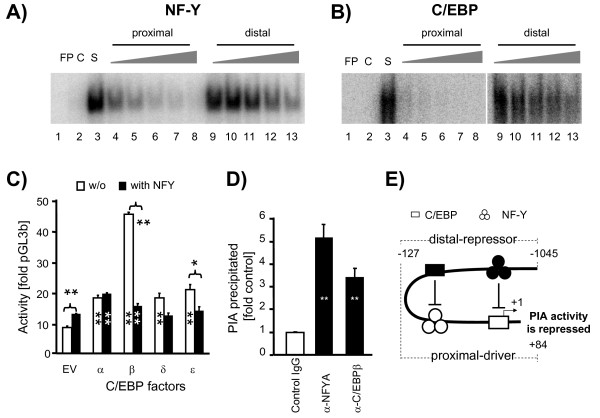
**Comparison of the binding affinities of the distal and proximal NF-Y and C/EBP sites and influence of these factors on the PIA activity.** (**A**) Affinity comparison of the proximal and distal NF-Y binding sites of PIA. Nuclear extracts were prepared from HEK293 cells jointly over-expressing the three bovine NF-Y factors (A, B, C). Sequences of the labeled NF-Y consensus probe and of the unlabeled competitor of the distal NF-Y (A_WT) and proximal NF-Y sites (pNF_WT) are shown in Additional file 1: Table S4 *cf* (Figures 2A and Figure 3A). Lane: FP, free probe; C, competition with 100-fold molar excess of unlabeled probe (self); S, shift. The fold molar excess of the respective unlabeled competitors added to the reactions, as resolved in lanes 4–8 or lanes 9–13 were 10, 25, 50, 100, 200-fold of the labeled probe. (**B**) Same as A, but evaluating the C/EBP binding sites. These samples were run out on the same gel. Nuclear extracts were prepared from HC-11 cells over-expressing the DNA binding domain of the bovine C/EBPβ factor. Sequences of the labeled C/EBP consensus probe, the unlabeled oligonucleotide competitors of C/EBP distal (C_WT) and C/EBP proximal (pCE_WT) are shown in Additional file 1: Table S4 *cf* (Figure 2A and Figure 3A). (**C**) Effects of NF-Y and C/EBPβ factors on the proximal PIA activity. The proximal PIA reporter construct −127 was transfected into HC-11 cells, together with the empty cloning vector (EV) or with constructs expressing C/EBPβ or NF-Y subunits, as indicated. The functional NF-Y trimer can quench this effect. Results (±S.E.M.) are representative for two experiments, each assayed in triplicate. Statistical significance of the differences was calculated relative to the control having been cotransfected only with empty vector (grey column), **P <0.01. (**D**) NF-Y factors interact with C/EBPβ having bound to PIA. HEK293 cells were co-transfected with the −127 PIA reporter gene and expression constructs for the factors C/EBPβ (tagged with a flag), NF-YAm29, -YB, -YC. Nuclei were used for ChIP assays using antibodies against C/EBPβ or NF-YA or control IgG. Data are mean values (±S.E.M.) of four qPCR assays of the amount of recovered PIA promoter molecules (**P <0.01, as compared to control IgG). (**E**) A master control unit modulates PIA activity. NF-Y and C/EBP factors (black) having bound to the distal repressive factor binding sites may be looped back to physically contact the respective other factor (open) having bound to the proximal driver region. This interaction lowers PIA activity. The map is not drawn to scale. “+1” represents the tsp.

The data presented the paradox that NF-Y as well as C/EBP-factors may either function as repressor or as driver of PIA activity, depending on binding to either the distal or the proximal sites on the promoter. We realized, however, in the course of the EMSA analyses that the binding affinities of the distal and the proximal sites were different. To quantitatively evaluate the differential binding affinities of the distal and proximal DNA-sequence motifs for both, NF-Y and C/EBPβ, we labeled as probes double stranded oligonucleotides harboring the core consensus motifs for either factor and embedded these into DNA-sequences which were otherwise entirely unrelated to the ACC-α PIA (NF-Y consensus, C/EBP consensus oligonucleotides, Additional file 1: Table S4). However, we choose as unlabeled competitors double stranded oligonucleotides with sequences representing either the distal or the proximal binding sites for the respective factors (for NF-Y: oligonucleotides A_WT, and pNF_WT; for C/EBP: oligonucleotides C_WT, pNF_WT, respectively; Additional file 1: Table S4). We used nuclear extracts for the assays prepared from cells overexpressing either all three NF-Y factors, or the factor C/EBPβ. Regarding the proximal NF-Y binding site (Figure 4A), we found that a 25-fold molar excess of the competitor would reduce the amount of the shifted probe down to 20% (Figure 4A, lane 5 and Additional file 1: Figure S3). In stark contrast, it required as much as a 200-fold molar excess of the distal attachment site to obtain the same degree of competition for the probe (Figure 4A, lane 13 and Additional file 1: Figure S3). An even larger difference in competition efficiency was observed for the C/EBP attachment sites (Figure 4B). As little as a 10-fold molar excess of the proximal site competitor reduced the shifted amount down to less than 20% (Figure 4B, lane 4 and Additional file 1: Figure S3). However, even a 200-fold molar excess of the distal site would still leave behind ~40% of the radioactive probe in the shifted band (Figure 4B, lane 13 and Additional file 1: Figure S3). These data demonstrate that the distal attachment sites have much lower binding affinities for the respective factors than thoseproximal sites.

### NF-Y quenches the stimulating effect of C/EBPβ

We analyzed how NF-Y and C/EBPβ together might repress PIA. Therefore, we co-transfected both factors either alone or jointly together and recorded the effect on the activity of the short PIA promoter segment. Both factors could separately activate the PIA reporter gene activity (Figure 4C and Additional file 1: Figure S4B). Co-transfection of expression constructs for all three NF-Y factors abolished the stimulatory effect of C/EBPβ (Figure 4C, Additional file 1: Figure S4A and Additional file 1: Figure S4B). Quenching of the C/EBPβ associated transactivation potential is dosage-dependent, since increasing amounts of transfected DNA caused stronger quenching of the stimulatory capacity of C/EBPβ (Additional file 1: Figure S4A). Quenching required that all three NF-Y subunits (A, B, C) were jointly expressed (Additional file 1: Figure S4B). We also found that NF-Y must not necessarily bind to the DNA to reduce the transactivating potential of C/EBPβ. This was shown by replacing three amino acids in the factor NF-YA. This replacement is known to abolish DNA binding of the NF-Y complex [26]. We found that inclusion of this mutated NF-YAm29 subunit was as efficiently quenching the C/EBPβ mediated PIA stimulation as the wild type NF-YA subunit (Additional file 1: Figure S4B).

The observation that the NF-Y factors might exert in trans their blocking effect on the C/EBPβ mediated activation of PIA was experimentally augmented. We expressed together in HEK293 cells the factors NF-YAm29, NF-YB, NF-YC and C/EBPβ and cotransfected the short PIA reporter gene. We analyzed from the same cells in ChIP assays if NF-Y factors may physically interact with C/EBPβ having bound to PIA. To this end, we measured in qPCR the amount of promoter molecules recovered by antibodies directed against C/EBPβ and NF-YA. We choose PCR amplification primers spanning the C/EBP site, but not the NF-Y binding site. We found that the antibodies against both factors retrieve approximately similar amounts of PIA (Figure 4D). These data show that NF-Y factors can indeed bind in trans to the C/EBP factor.

### Different tissue-specific levels of both, PIA activity and C/EBPβ mRNA concentrations

We examined if our in vitro derived data concerning mechanism regulating PIA activity are of physiological relevance in vivo. We exploited for this purpose tissue samples derived from two different stacks of animals. Livers were taken from those 12 lactating cows having been fed normal and energy reduced straw diets, having above already been referred to. In addition, adipose and mammary gland tissues were collected from 16 mid lactating cows having been fed 30 days before slaughter either a norm diet or a fat enriched diet. Experimental conditions for those two groups of animals have extensively been described [28,30].

We compared the PIA activity in different tissues to the mRNA abundance of both families of transcription factors. The concentration of PIA derived transcripts differed >100-fold between liver and lactating mammary gland, while intermediate concentrations were found in adipose tissue (Table1). This indicates substantial tissue-specific differences in PIA activity. The concentration of NF-YA-encoding mRNA molecules was fairly constant. However, the concentrations of C/EBPβ varied between the tissues. Analysis of a potential interdependence between the mRNA concentrations of those factors revealed a strikingly clear correlation ofPIA derived transcripts with the ratio between the tissue-specific mean values of NF-YA mRNA copies over those of C/EBPβ (Additional file 1: Figure S5). The correlation between these parameters is statistically high and significant (full symbols; r, 0.997; p < 0.001). Moreover, including the mean values derived from the different nutritional subgroups (Table1) into this correlation analysis reveals that the nutrition-dependent different mean values also fit very well to this correlation (open symbols, Additional file 1: Figure S5). However, due to the eventually large individual variation, not all differences between the subgroup means of a given tissue are statistically significant cf (Table1). No significant degree of correlation was found if all the individual data were considered, due to the high degree of individual variation. We found the highest degree of individual variability for the levels of PIA derived transcripts (CV, 116), while the same parameter was much lower for the mRNA concentrations of those transcription factors (52% and 5%, for C/EBPβ and NF-YA respectively).

The mRNA concentrations of both factors are very different in the respective model cells. We found in those pbMEC cultures from cows ~40fold higher concentrations of the NF-YA (3.1 +0.2 x 10^5^, n = 3) and only ~1% of the C/EBPβ mRNA concentration (5.4 + 0.32 x 10^3^, n = 3) than found in vivo in udder tissue. This very low endogenous concentration of the C/EBPβ mRNA in the pbMEC model cells may relate to the observation that overexpression of the C/EBPβ mRNA encoding vector in these cells significantly increased the activity of the long PIA-reporter construct. This would not be predicted by the above model. The C/EBPβ mRNA concentration in pbMEC is very similar to that previously reported from us for the murine HC-11 MEC model cell [11]. Neither of these model cells endogenously expresses PIA.

## Discussion

We initiated this study in order to characterize the molecular principle repressing the general activity of PIA of the ACC-α-encoding gene in cattle. To ensure the physiological relevance of our analysis, we first validated for the cow that PIA, indeed is the nutritionally regulated promoter. This was previously only assumed to be the case [7] by analogy with the situation found in other animals including rat [31,32]. We also excluded that a more upstream located ACC-α promoter “PI” (EMBL Acc No FN185962) would override those transcriptional controls of PIA. We found this promoter to be prominently expressed in the brain (Shi, unpublished), just as that from sheep [10]. Significantly, transcripts derived from PI do not contain exon1A derived sequences, as revealed by 5´-RACE experiments (EMBL acc No FN185963). Thus, transcripts containing exon1A truly stem from PIA and thus are diagnostic for the activity of PIA.

### NF-Y and C/EBP factors may either repress or activate PIA activity, depending on the binding site in the promoter

Our mapping and mutation data indicate that structurally the bipartite PIA repressor is composed of attachment sites for CCAAT-box binding transcription factors of the NF-Y and of the C/EBP families, binding to the distal promoter. Their repressive strength is additive, at least. However, we also observed that attachment sites for both transcription factors on the proximal promoter function as activators for PIA activity. Hence, the binding position rather than the nature of the CCAAT-box binding factor itself determines its function as either to repress or to activate PIA activity.

The bipartite structure of the PIA repressor in cattle is similar to that of the rat PIA promoter [17]. The 5´-part of the rat PIA repressor is formed by a microsatellite, a CA28 repeat. It is not known which factor might bind there. The distal element of the bovine PIA repressor resides in an Art2 retroposon element [7]. We now found that NF-Y factors may bind there to repress PIA activity. The 3´-part of the repressor was found in rat [16,17] as well as in cattle (this study) to depend on a C/EBP factor binding site. Hence, the structurally divergent, yet physiologically equivalent PIA promoters of rat and cow feature a similar bipartite structure of that repressor. However, the structures of the 5´-segment of that repressor are completely unrelated between cattle and rat.

NF-Y is known to regulate the transcription of many genes by interacting with the CCAATbox sequence motif, either enhancing [22,33,34] or dampening expression [27,35]. Dual NFY binding sites of high and low binding affinities have been reported to reside in the promoter of the human von Willebrand factor (VWF) gene [36]. The low affinity site, located in exon1, was found to repress VWF promoter activity, while the high affinity site functions as an activator, quite similar to our findings in the cattle PIA promoter. Interestingly, the high affinity activator binding site of the human VWF gene spans the positions from −30 to +1, very comparable as found in the bovine PIA. The repressive function of NF-Y involves interaction with histone deacetylase (HDAC). NF-Y recruits HDACs to the VWF promoter thus inhibiting the promoter activity in non-endothelial cells. This repression depends on NFY-binding to that downstream repressive site [37].

Likewise, we found that two C/EBP binding sites are crucially involved in regulating the PIA promoter activity. The proximal binding site contributes to basal promoter activity. Stimulation of gene expression through C/EBP factors has often been found [38-40]. It has also been observed, that C/EBP factors may repress transcription [15,41], eventually through repressive family members, such as C/EBPγ and -ζ, or repressive isoforms [19] or through the interaction with HDAC in various physiology conditions [42]. Hence, also the C/EBP family of transcription factors is known to either positively or negatively regulate gene expression.

Our data show that two criteria determine whether these factors activate or repress PIA promoter activity: First, their binding to either the distal repressor or proximal driver region matters but also, second their eventual interaction.

### NF-Y represses the activating capability of C/EBPβ via protein-protein interaction

We observe, as a novel mechanism that the functional NF-Y factor complex reduces the strong enhancing effect of C/EBPβ by protein-protein interaction. The repressive effect of NF-Y on the C/EBPβ mediated activation may be unique to PIA of the ACC-α in cattle. In contrast to our observation, it was found in mouse that the interaction between NF-Y with C/EBPα strongly enhances the stimulatory effect on the amelogenin gene promoter activity. ChIP experiments indicated in this example that NF-Y interacts in trans with C/EBPα [23]. C/EBPδ could eventually substitute for the role of the C/EBPα factor [43]. Similarly, expression of the human microsomal-epoxide-hydrolase gene (EPHX) is stimulated through interaction of promoter bound NF-Y factors with C/EBPα. This interaction must be mediated in trans, since this promoter does not bind directly the factor C/EBPα [44].

We also found that protein-protein interaction mediates the repressive function of NF-Y factors on the stimulating effect of C/EBPβ, based on two lines of evidence. Reporter gene assays involving the mutated DNA-binding incompetent factor NF-YA_m29_ and ChIP data congruently support the conclusion that the repressive effect of NF-Y on C/EBPβ mediated enhancement of PIA activity is mediated in trans.

Our data may therefore be summarized in a model of a master control unit regulating PIA activity (Figure 4E). Small amounts of NF-Y and C/EBPβ factors are sufficient to saturate the proximal binding sites and to activate the promoter. Hence, their high binding affinity for those factors ensures that PIA in principal could actively transcribe the gene, almost in any tissue given that both transcription factors are ubiquitously present. Indeed, a pivotal role of C/EBPβ in lipid synthesis has recently been proven by showing that functional disruption of the C/EBPβ-encoding gene results in impaired fat accumulation in adipose tissue [41].

However, recruitment of a transcription factor to a high affinity site cannot reflect different factor concentrations, since high affinity sites respond similarly to a switch (e.g. either “on” or “off”) rather than like an adjustable lever. To nevertheless achieve regulation, PIA activity is thus dampened by the factors binding to those distal, low affinity sites. Much higher factor concentrations are necessary to saturate them. Hence, the probability that they are occupied can reflect different factor concentrations. The very large difference observed between the binding affinities of the proximal vs distal C/EBP binding sites suggests that these might be of pivotal significance in this regard. Once those distal, repressive sites are occupied, they might physically be brought into contact with the reciprocal factor residing on the proximal promoter to repress via protein-protein interaction the activation potential. This would structurally require that the promoter elements separating the different components of the master control unit are looped out.

### C/EBPβ may be the prominent factor to constrain the tissue-specific PIA activity

PIA activity, as assessed on the basis of PIA derived transcripts differs significantly between tissues, similar as it is found for the balance between the concentrations of NF-YA and C/EBPβ-encoding mRNA molecules. The latter ratio is mainly influenced by large tissue-specific differences of the C/EBPβ mRNA concentration. Assuming that the mRNA concentrations reflect the factor concentrations we then observe, in keeping with our model the highest PIA activity in the lactating mammary glands, where also the C/EBPβ mRNA concentrations (e.g. factor concentrations) are least (Table1). Moreover, the diet dependent changes of PIA activity in this gland are inversely correlated with the C/EBPβ mRNA concentrations. The average PIA activity is highest in the mammary gland of cows having been fed with a fat-enriched diet and concomitantly we found that the C/EBPβ mRNAconcentrations are least under these conditions. C/EBPβ is also found to be regulated in the mammary gland of the mouse by the fat content of the diet [29], as well as with different stages of lactation [45].

Taken together our data suggest that tissue-specific alterations in the proportion of NF-Y and C/EBPβ adjust very different set points for the gross PIA activity around which other factors may finely tune the individual PIA activity according to the acute actual needs.

### Model cells do not properly reflect the situation in the tissues

It would have been desirable to validate this model through manipulation of the ratio of NF-Y / C/EBPβ factors. However, the initial mRNA concentrations for both factors are so vastly different in the pbMEC cells compared to the situation found in the udder tissue that a direct comparison is not really meaningful. Indeed, it is long known that these pbMEC express neither genes for milk protein- nor milk fat-synthesis [46]. They also do not express the endogenous ACC-α PIA (own measurements, data not shown). Moreover, we previously attempted to establish primary cultures of liver cells from cattle as relevant cell model for the analysis of PIA regulation. We found that within 1 h after extirpation the cells would only feature 10% of the original PIA mRNA concentration and another 2.5 h later this concentration was barely measurable, down to ~3% of the original mRNA concentration.

Hence, these cell models do not maintain and reflect the developmentally finely tuned situation found in vivo in the specific tissue within the organ.

## Conclusions

The proximal promoter segment of PIA appears to be principally in an active state, since even minute concentrations of both, NF-Y and C/EBPβ factors can saturate the high affinity activator sites. Higher factor concentrations will saturate the low affinity repressive sites on the distal promoter resulting in reduced and calibrated promoter activity. Based on measurements of the mRNA concentrations of those factors in different tissues we propose that the interplay of both factors may set tissue-specific limits for PIA activity.

## Methods

### Animals

All animal experiments were performed according to protocols approved by the relevant authorities (Landesamt für Landwirtschaft, Lebensmittelsicherheit und Fischerei, Mecklenburg-Vorpommern).

### Reporter gene constructs

All oligonucleotides used throughout the study are listed in Additional file 1: Table S1. Establishment of the constructs in pGL3basic (Promega) is described in Additional file 2: Supplementary materials and methods.

### Fusion PCR

Fusion PCR was performed as described [47]. All primers are listed in Additional file 1: Table S2, and a detailed protocol is given in Additional file 2: Supplementary method.

### Cell culture, transient transfection and luciferase assays

Cells from the established murine mammary epithelial cell line (HC-11), primary bovine Mammary Epithelial Cells (pbMEC) and human Embryonic Kidney Cells (HEK293) were cultured as previously described [7,48]. Transient transfection and luciferase assays were done essentially as described [7]. Co-transfection of the vector phRL-TK renilla (Promega) allowed to eventually normalizing for slightly varying transfection efficiencies. The normalized data were expressed as multiples of the activity of the promoterless pGL3basic clone, the hosting vector for the different promoter fragments.

### Quantitative real-time PCR

The abundances of ACC-α transcripts derived from PIA, PII, or from all promoters (total), and those encoding NF-YA and C/EBPβ were measured by quantitative Real-time PCR (RT-qPCR) assays as described [7,8]. Sampling of the biopsies from liver, mammary gland and fat was already described [28]. Tissues were snap-frozen in liquid nitrogen immediately after collection. One hundred milligram of ground tissue in 1 ml of TRIzol reagent (Invitrogen) was homogenized with Ultra-Turrax T25 for 10 s. After addition of 0.2 ml of chloroform and centrifugation at 12,000 g for 15 min at 4°C, aqueous phase was transferred for total RNA precipitation by adding 1 volume of isopropanol. The RNA pellet was washed with 1 ml of 70% ethanol and air-dried. RNA was dissolved in DEPC-treated water and stored at −70°C. RNA quality was analyzed by agarose gel electrophoresis containing formaldehyde and RNA concentration was measured with a Nanodrop spectrophotometer. Two μg of total RNA was first primed in reverse with oligo (dT)_20_ and a gene specific primer (primers listed in Additional file 1: Table S3)using Superscript II reverse transcriptase (Invitrogen) as recommended by supplier. The first-strand cDNA was purified with the High Pure PCR Product Purification Kit (Roche) and eluted with 100 μl of water. Real-time PCR was carried out with the LightCyclerFastStart DNA Master SYBR Green I Kit (Roche). The 10 μl assays contained 5 μl of cDNA template, 2.6 μl of water, 0.2 μl of each 25 μM forward and reverse primers and 2 μl of the master mix. Pre-incubation was performed at 95°C for 10 min, and amplification was carried out as 95°C 15s; 60°C 5s; 72°C 20s for 40 cycles. Primers and amplicon sizes are described in Additional file 1: Table S3. Beta-actin was used to normalize transcription data. A dilution series (10^1^-10^6^copies) of the appropriate cDNA subclones were included in each run and served as an external standard to calculate the copy numbers of transcripts. All assays were run in duplicate.

### Electrophoretic mobility shift assay

Preparation of nuclear extracts and Electrophoretic Mobility Shift Assays (EMSA) were performed as described [48]. Probes were labeled radioactively with ^32^P-dCTP using the Klenow-fill in procedure, as described [9]. To this end, we primed the fill-in reaction on the respective “long” oligonucleotide (Additional file 1: Table S4) by annealing the corresponding “short” reverse oligonucleotide. Labeled probes and nuclear extracts were incubated with 10 μl of 2x binding buffer (20 mM HEPES pH7.9, 10 mM MgCl_2_, 0.2 mM EDTA pH8, 2 mM DTT, 0.1 M NaCl, 20% Glycerol, 0.4 mM PMSF) in 20 μl of volume. DNA-protein complexes were separated on 4% or 6% (NF-Y or C/EBP, respectively) non-denaturing polyacrylamide gels. Anti-NF-YA (sc-10779x) and anti-C/EBPβ (sc-746x) antibodies were purchased from Santa Cruz Biotechnology (Santa Cruz, CA, USA).

### Chromatin Immunoprecipitation assays

The general procedures for chromatin immunoprecipitation assays (ChIP) were essentially as described [49]. Details of the experiment are shown in Additional file 2: Supplementary methods.

### Statistical analysis

The statistical significance of differing mean values was assessed with Student’s t-test, as provided with the Excel program by the Microsoft Office software.

## Abbreviations

ACC, α (eg greek alpha)acetyl-CoA carboxylase-alpha; PIA, promoter IA; C/EBP, CCAAT/enhancer binding protein; NF-Y, Nuclear factor-Y; EMSA, Electrophoretic mobility shift assay; qPCR, Quantitative Real-time PCR; ChIP, Chromatin immunoprecipitation; HC-11, Murine mammary epithelial cells; HEK293, Human embryonic kidney cells.

## Competing interests

The authors declare that there are not any financial or non-financial competing interests associated with this work.

## Authors’ contributions

XS conducted the molecular biology experiments, has contributed developing the design of the study and drafted the ms. CM contributed to designing the study and the animal experimentation. HMS initiated and supervised the work and finalized the manuscript. All authors read and approved the final manuscript.

## Supplementary Material

Additional file 1 Supplementary Figures and Tables. Click here for file

Additional file 2 **Supplementary Material and methods.**[7,47,50-52]. Click here for file

## References

[B1] Kim KH Regulation of mammalian acetyl-coenzyme A carboxylase Annu Rev Nutr 1997 17 77 99 10.1146/annurev.nutr.17.1.77 9240920

[B2] Mao J Chirala SS Wakil SJ Human acetyl-CoA carboxylase 1 gene: Presence of three promoters and heterogeneity at the 5'-untranslated mRNA region Proc Natl Acad Sci USA 2003 100 7515 7520 10.1073/pnas.1332670100 12810950 PMC164618

[B3] Brusselmans K De Schrijver E Verhoeven G Swinnen JV RNA Interference-Mediated Silencing of the Acetyl-CoA-Carboxylase-α Gene Induces Growth Inhibition and Apoptosis of Prostate Cancer Cells Cancer Res 2005 65 6719 6725 10.1158/0008-5472.CAN-05-0571 16061653

[B4] Wakil SJ Abu-Elheiga LA Fatty acid metabolism: target for metabolic syndrome J Lipid Res 2009 50 S138 S143 19047759 10.1194/jlr.R800079-JLR200 PMC2674721

[B5] Loor JJ Everts RE Bionaz M Dann HM Morin DE Oliveira R Nutrition-induced ketosis alters metabolic and signaling gene networks in liver of periparturient dairy cows Physiol Genomics 2007 32 105 116 10.1152/physiolgenomics.00188.2007 17925483

[B6] Barber MC Price NT Travers MT Structure and regulation of acetyl-CoA carboxylase genes of metazoa Biochim Biophys Acta 2005 1733 1 28 10.1016/j.bbalip.2004.12.001 15749055

[B7] Mao J Marcos S Davis SK Burzlaff J Seyfert HM Genomic distribution of three promoters of the bovine gene encoding acetyl-CoA carboxylase alpha and evidence that the nutritionally regulated promoter I contains a repressive element different from that in rat Biochem J 2001 358 127 135 10.1042/0264-6021:3580127 11485560 PMC1222040

[B8] Mao J Seyfert HM Promoter II of the bovine acetyl-coenzyme A carboxylase-alpha-encoding gene is widely expressed and strongly active in different cells Biochim Biophys Acta 2002 1576 324 329 10.1016/S0167-4781(02)00369-X 12084579

[B9] Mao J Molenaar AJ Wheeler TT Seyfert HM STAT5 binding contributes to lactational stimulation of promoter III expressing the bovine acetyl-CoAcarboxylase alpha-encoding gene in the mammary gland J Mol Endocrinol 2002 29 73 88 10.1677/jme.0.0290073 12200230

[B10] Travers MT Cambot M Kennedy HT Lenoir GM Barber MC Joulin V Asymmetric expression of transcripts derived from the shared promoter between the divergently oriented ACACA and TADA2L genes Genomics 2005 85 71 84 10.1016/j.ygeno.2004.10.001 15607423

[B11] Shi X Liu S Metges CC Seyfert HM C/EBP-beta drives expression of the nutritionally regulated promoter IA of the acetyl-CoA carboxylase-alpha gene in cattle Biochim Biophys Acta 2010 1799 561 567 10.1016/j.bbagrm.2010.07.002 20637321

[B12] Molenaar A Mao J Oden K Seyfert HM All Three Promoters of the Acetyl-Coenzyme A-Carboxylase {alpha}-encoding Gene Are Expressed in Mammary Epithelial Cells of Ruminants J Histochem Cytochem 2003 51 1073 1081 10.1177/002215540305100811 12871989

[B13] Lopez-Casillas F Kim KH The 5' untranslated regions of acetyl-coenzyme A carboxylase mRNA provide specific translational control in vitro Eur J Biochem 1991 201 119 127 10.1111/j.1432-1033.1991.tb16264.x 1680679

[B14] Travers MT Vallance AJ Gourlay HT Gill CA Klein I Bottema CB Promoter I of the ovine acetyl-CoA carboxylase-alpha gene: an E-box motif at −114 in the proximal promoter binds upstream stimulatory factor (USF)-1 and USF-2 and acts as an insulin-response sequence in differentiating adipocytes Biochem J 2001 359 273 284 10.1042/0264-6021:3590273 11583573 PMC1222145

[B15] O'Callaghan BL Koo SH Wu Y Freake HC Towle HC Glucose Regulation of the Acetyl-CoA Carboxylase Promoter PI in Rat Hepatocytes J Biol Chem 2001 276 16033 16039 10.1074/jbc.M101557200 11340083

[B16] Tae HJ Zhang S Kim KH cAMP Activation of CAAT Enhancer-binding Protein-beta Gene Expression and Promoter I of Acetyl-CoA Carboxylase J Biol Chem 1995 270 21487 21494 10.1074/jbc.270.37.21487 7545164

[B17] Tae HJ Luo X Kim KH Roles of CCAAT/enhancer-binding protein and its binding site on repression and derepression of acetyl-CoA carboxylase gene J Biol Chem 1994 269 10475 10484 7908293

[B18] McKnight SL Lane MD Gluecksohnwaelsch S Is Ccaat/Enhancer-Binding Protein A Central Regulator of Energy-Metabolism Genes Dev 1989 3 2021 2024 10.1101/gad.3.12b.2021 2697636

[B19] Schrem H Klempnauer J Borlak J Liver-Enriched Transcription Factors in Liver Function and Development. Part II: the C/EBPs and D Site-Binding Protein in Cell Cycle Control, Carcinogenesis, Circadian Gene Regulation, Liver Regeneration, Apoptosis, and Liver-Specific Gene Regulation Pharmacol Rev 2004 56 291 330 10.1124/pr.56.2.5 15169930

[B20] Descombes P Schibler U A Liver-Enriched Transcriptional Activator Protein, Lap, and A Transcriptional Inhibitory Protein, Lip, Are Translated from the Same Messenger-Rna Cell 1991 67 569 579 10.1016/0092-8674(91)90531-3 1934061

[B21] Descombes P Chojkier M Lichtsteiner S Falvey E Schibler U Lap, A Novel Member of the C/Ebp Gene Family, Encodes A Liver-Enriched Transcriptional Activator Protein Genes Dev 1990 4 1541 1551 10.1101/gad.4.9.1541 2253878

[B22] Park Sk Oh SY Lee MY Yoon S Kim KS Kim Jw CCAAT/Enhancer Binding Protein and Nuclear Factor-Y Regulate Adiponectin Gene Expression in Adipose Tissue Diabetes 2004 53 2757 2766 10.2337/diabetes.53.11.2757 15504955

[B23] Xu Y Zhou YL Luo W Zhu QS Levy D MacDougald OA NF-Y and CCAAT/Enhancer-binding Protein α Synergistically Activate the Mouse Amelogenin Gene J Biol Chem 2006 281 16090 16098 10.1074/jbc.M510514200 16595692

[B24] Tomita T Kimura S Regulation of mouse Scgb3a1 gene expression by NF-Y and association of CpG methylation with its tissue-specific expression BMC Mol Biol 2008 9 5 10.1186/1471-2199-9-5 18194566 PMC2266941

[B25] Gurtner A Manni I Fuschi P Mantovani R Guadagni F Sacchi A Requirement for down-regulation of the CCAAT-binding activity of the NF-Y transcription factor during skeletal muscle differentiation Mol Biol Cell 2003 14 2706 2715 10.1091/mbc.E02-09-0600 12857858 PMC165670

[B26] Mantovani R Li XY Pessara U Hooft van Huisjduijnen R Benoist C Mathis D Dominant negative analogs of NF-YA J Biol Chem 1994 269 20340 20346 8051128

[B27] Bernadt CT Nowling T Wiebe MS Rizzino A NF-Y behaves as a bifunctional transcription factor that can stimulate or repress the FGF-4 promoter in an enhancer-dependent manner Gene Expr 2005 12 193 212 10.3727/000000005783992052 16128003 PMC6009113

[B28] Kuhla B Albrecht D Kuhla S Metges CC Proteome analysis of fatty liver in feed-deprived dairy cows reveals interaction of fuel sensing, calcium, fatty acid, and glycogen metabolism Physiol Genomics 2009 37 88 98 10.1152/physiolgenomics.90381.2008 19240300

[B29] Rudolph MC McManaman JL Phang T Russell T Kominsky DJ Serkova NJ Metabolic regulation in the lactating mammary gland: a lipid synthesizing machine Physiol Genomics 2007 28 323 336 17105756 10.1152/physiolgenomics.00020.2006

[B30] Duske K Hammon HM Langhof AK Bellmann O Losand B Nurnberg K Metabolism and lactation performance in dairy cows fed a diet containing rumen-protected fat during the last twelve weeks of gestation J Dairy Sci 2009 92 1670 1684 10.3168/jds.2008-1543 19307649

[B31] Lopezcasillas F Poncecastaneda MV Kim KH Invivo Regulation of the Activity of the 2 Promoters of the Rat Acetyl Coenzyme-A Carboxylase Gene Endocrinology 1991 129 1049 1058 10.1210/endo-129-2-1049 1677328

[B32] Travers MT Barber MC Tissue-specific control of the acetyl-CoA carboxylase gene Biochemical Soc Tran 1997 25 1215 1219 10.1042/bst0251215 9449978

[B33] Borghini S Vargiolu M Di Duca M Ravazzolo R Ceccherini I Nuclear Factor Y Drives Basal Transcription of the Human TLX3, a Gene Overexpressed in T-Cell Acute Lymphocytic Leukemia Mol Cancer Res 2006 4 635 643 10.1158/1541-7786.MCR-05-0250 16966433

[B34] Matsuo N Yu-Hua W Sumiyoshi H Sakata-Takatani K Nagato H Sakai K The Transcription Factor CCAAT-binding Factor CBF/NF-Y Regulates the Proximal Promoter Activity in the Human α1(XI) Collagen Gene (COL11A1) J Biol Chem 2003 278 32763 32770 10.1074/jbc.M305599200 12805369

[B35] Manni I Mazzaro G Gurtner A Mantovani R Haugwitz U Krause K NF-Y Mediates the Transcriptional Inhibition of the cyclin B1, cyclin B2, and cdc25C Promoters upon Induced G2 Arrest J Biol Chem 2001 276 5570 5576 10.1074/jbc.M006052200 11096075

[B36] Peng YW Jahroudi N The NFY transcription factor functions as a repressor and activator of the von Willebrand factor promoter Blood 2002 99 2408 2417 10.1182/blood.V99.7.2408 11895773

[B37] Peng Y Stewart D Li W Hawkins M Kulak S Ballermann B Irradiation modulates association of NF-Y with histone-modifying cofactors PCAF and HDAC Oncogene 2007 26 7576 7583 10.1038/sj.onc.1210565 17599060

[B38] Reddy KV Serio KJ Hodulik CR Bigby TD 5-lipoxygenase-activating protein gene expression - Key role of CCAAT/enhancer-binding proteins (C/EBP) in constitutive and tumor necrosis factor (TNF) alpha-induced expression in THP-1 cells J Biol Chem 2003 278 13810 13818 10.1074/jbc.M211102200 12571239

[B39] Zhang F Lin M Abidi P Thiel G Liu J Specific Interaction of Egr1 and c/EBPβ Leads to the Transcriptional Activation of the Human Low Density Lipoprotein Receptor Gene J Biol Chem 2003 278 44246 44254 10.1074/jbc.M305564200 12947119

[B40] Zhou YL Snead ML Identification of CCAAT/Enhancer-binding Protein alpha as a Transactivator of the Mouse Amelogenin Gene J Biol Chem 2000 275 12273 12280 10.1074/jbc.275.16.12273 10766866

[B41] Schroeder-Gloeckler JM Rahman SM Janssen RC Qiao L Shao J Roper M CCAAT/Enhancer-binding Protein beta Deletion Reduces Adiposity, Hepatic Steatosis, and Diabetes in Leprdb/db Mice J Biol Chem 2007 282 15717 15729 10.1074/jbc.M701329200 17387171 PMC4109269

[B42] Wang GL Salisbury E Shi XR Timchenko L Medrano EE Timchenko NA HDAC1 cooperates with C/EBP alpha in the inhibition of liver proliferation in old mice J Biol Chem 2008 283 26169 26178 10.1074/jbc.M803544200 18622015 PMC2533775

[B43] Xu Y Zhou YL Gonzalez FJ Snead ML CCAAT/enhancer-binding protein delta (C/EBPdelta) maintains amelogenin expression in the absence of C/EBPalpha in vivo J Biol Chem 2007 282 29882 29889 10.1074/jbc.M702097200 17704518 PMC4445686

[B44] Zhu QS Qian B Levy D CAAT/Enhancer-binding Protein α (C/EBPα) Activates Transcription of the Human Microsomal Epoxide Hydrolase Gene (EPHX1) through the Interaction with DNA-bound NF-Y J Biol Chem 2004 279 29902 29910 10.1074/jbc.M400438200 15150264

[B45] Gigliotti AP DeWille JW Lactation status influences expression of CCAAT/enhancer binding protein isoform mRNA in the mouse mammary gland J Cell Physiol 1998 174 232 239 10.1002/(SICI)1097-4652(199802)174:2<232::AID-JCP10>3.0.CO;2-E 9428809

[B46] Günther J Koczan D Yang W Nürnberg G Repsilber D Schuberth HJ Park Z Maqbool N Molenaar A Seyfert HM Assessment of the immune capacity of mammary epithelial cells: comparison with mammary tissue after challenge with Escherichia coli Vet Res 2009 40 31 10.1051/vetres/2009014 19321125 PMC2695127

[B47] Liu S Shi X Bauer I Gunther J Seyfert HM Lingual antimicrobial peptide and IL-8 expression are oppositely regulated by the antagonistic effects of NF-kappaB p65 and C/EBPbeta in mammary epithelial cells Mol Immunol 2011 48 895 908 10.1016/j.molimm.2010.12.018 21255844

[B48] Yang W Molenaar A Kurts-Ebert B Seyfert HM NF-kappa B factors are essential, but not the switch, for pathogen-related induction of the bovine beta-defensin 5-encoding gene in mammary epithelial cells Mol Immunol 2006 43 210 225 10.1016/j.molimm.2005.02.003 16199258

[B49] Zhan X Shi X Zhang Z Chen Y Wu JI Dual role of Brg chromatin remodeling factor in Sonic hedgehog signaling during neural development Proc Natl Acad Sci USA 2011 108 12758 12763 10.1073/pnas.1018510108 21768360 PMC3150941

[B50] Nowak DE Tian B Brasier AR Two-step cross-linking method for identification of NF-kappa B gene network by chromatin immunoprecipitation Biotechniques 2005 39 715 725 10.2144/000112014 16315372

[B51] Zhao JQ Glasspool RM Hoare SF Bilsland A Szatmari I Keith WN Activation of telomerase rna gene promoter activity by NF-Y, Sp1, and the retinoblastoma protein and repression by Sp3 Neoplasia 2000 2 531 539 10.1038/sj.neo.7900114 11228546 PMC1508088

[B52] Wu J Metz C Xu X Abe R Gibson AW Edberg JC Cooke J Xie F Cooper GS Kimberly RP A Novel Polymorphic CAAT/Enhancer-Binding Protein beta Element in the FasL Gene Promoter Alters Fas Ligand Expression: A Candidate Background Gene in African American Systemic Lupus Erythematosus Patients J Immunol 2003 170 132 138 12496392 10.4049/jimmunol.170.1.132

