# Supplementary material for: Interaction of C/EBP-beta and NF-Y factors constrains activity levels of the nutritionally controlled promoter IA expressing the acetyl-CoA carboxylase-alpha gene in cattle
Source: BMC Mol Biol. 2012 Jun 27;13:21. doi: 10.1186/1471-2199-13-21 (PMC3441787; doi:10.1186/1471-2199-13-21)
Supplement: Additional file 1 — Supplementary Figures and Tables. [file 1471-2199-13-21-S1.pdf]

Supplementary Material and methods

Reporter gene constructs:

Establishment of the constructs featuring the long wild type PIA reporter (-1045), deletion of element A (ΔA), of the central segment between the *Stu*I sites (ΔStu) and the short proximal promoter (-127) have previously been described [1]. Vector ΔNde was constructed by digesting clone -1045 with *Nde*I and subsequent religation. PCR amplification was used to construct clone ΔB, using primer pair fa1/rb (Tab. S1) and clone ΔNde as a template. We used oligonucleotides to eventually introduce unique restriction sites, to facilitate subsequent cloning steps. The PCR product (from -1045 to –828) was digested with *Kpn*I/*Nde*I and cloned into the respective sites of clone ΔNde. To construct clones ΔC1 and ΔC2, amplifications were conducted with the element C-specific forward primer (fc1 and fc2 respectively) introducing 5´- a *Nde*I site and the reverse primer Luc_r attaching to the 5´-part of the firefly luciferase gene in pGL3basic (pGL3b, Promega). The amplicons were digested with *Nde*I/*Hind*III and cloned into the respective sites of clone ΔNde. Clone ΔA-L was constructed based on clone -1045 by PCR. Amplification was performed with the forward primer fa2 and the reverse primer Luc_r. The PCR product was blunted (Klenow fill-in) and digested with *Hind*III, subsequently cloned into the *Kpn*I (blunted with Klenow)/*Hind*III sites of clone -1045. Clone ΔA-R was constructed by Fusion PCR as described below, using clone -1045 as a template. The amplicon was digested with *Kpn*I/*Hind*III and cloned into the respective sites of pGL3b.

***Fusion PCR*:**

Fusion PCR was performed as described [2]. All primers are listed in Tab. S2. Primer1 (P1, f) and Primer2 (P2, r) were derived from pGL3basic, upstream and downstream sequences of the multi-cloning site, respectively. The mutation primer pairs of Primer3 (P3) and Primer4 (P4) were devised for either mutation or deletion. Amplifications using the primer pair P1/P3 generated product1, while product 2 was amplified with primer pair P2/P4. After gel purification, equal molar amounts of product1 and product2 were mixed together in a PCR reaction system suitable for subsequent touchdown PCR amplifications. After an initial denaturation (95°C, 5 min), the mixture was cycled 5 times (65°C [lowered by 1°C per cycle], 30 sec, 72°C, 2 min, 95°C, 30 sec). Next, primers P1 & P2 were added and another 30 cycles were conducted, using 60°C as annealing temperature. The fusion PCR products were cloned into the pGEM-T easy vector (Promega) and validated by sequencing. Mutated reporters Am, Ym, m1 and m2 were based on clone -1045 as template. Clone Cm1 was based on clone ΔNde. The respective amplicons were digested with *Kpn*I/*Hind*III enzymes and cloned into the respective sites of pGL3basic. To construct clone Ym, m1 and m2, the amplicons were digested with *Kpn*I and *Stu*I, blunted and religated. Clone ΔA-R deleted the right half of element A, from position -1022 to -998. Therefore, the 5´-end of primer P4:fAR annealed to position -1031 to -1023, while the 3´-half of it annealed from positions -997 to -978. All constructs were confirmed by sequencing.

Chromatin Immunoprecipitation Assays:

The general procedures for chromatin immunoprecipitation assays (ChIP) were essentially as described [3]. HEK293 cells were co-transfected with 4 μg of the short PIA reporter construct (-127), and different amounts of expression constructs for the factors C/EBPβ (4 μg), NF-YAm (8 μg), NF-YB (8 μg) and NF-YC (4 μg). The transfected cells were first fixed with 2 mM disuccinimidyl glutarate (DSG, 45 min) and subsequently with 1 % formaldehyde

(10 min), as suggested [4]. The set of reagents for ChIP experiments was from Upstate Biotechnology. The sheared chromatin was precleared with 75 µl of protein-A agarose beads for 2 h, and subsequently by incubating for another two h with 10 µg of normal rabbit IgG (sc-2027, Santa Cruz Biotechnology). These IgG were removed with Protein A agarose beads. Next, four micrograms of antibodies against C/EBPβ, NF-YA, or normal rabbit IgG were used for ChIP assays. After extensive washes and elution, the amount of PIA promoter molecules recovered by this procedure was quantified by qPCR. The primers were placed such to amplify the region containing the proximal C/EBP site of PIA. Primers sequences are listed in Tab. S3.

References

1. Mao J, Marcos S, Davis SK, Burzlaff J, Seyfert HM: **Genomic distribution of three promoters of the bovine gene encoding acetyl-CoA carboxylase alpha and evidence that the nutritionally regulated promoter I contains a repressive element different from that in rat**. *Biochem J* 2001, **358:**127-135.

2. Liu S, Shi X, Bauer I, Gunther J, Seyfert HM: **Lingual antimicrobial peptide and IL-8 expression are oppositely regulated by the antagonistic effects of NF-kappaB p65 and C/EBPbeta in mammary epithelial cells**. *Mol Immunol* 2011, **48:**895-908.

3. Zhan X, Shi X, Zhang Z, Chen Y, Wu JI: **Dual role of Brg chromatin remodeling factor in Sonic hedgehog signaling during neural development**. *Proc Natl Acad Sci U S A* 2011, **108:**12758-12763.

4. Nowak DE, Tian B, Brasier AR: **Two-step cross-linking method for identification of NF-kappa B gene network by chromatin immunoprecipitation**. *Biotechniques* 2005, **39:**715-725.

5. Zhao JQ, Glasspool RM, Hoare SF, Bilsland A, Szatmari I, Keith WN: **Activation of telomerase rna gene promoter activity by NF-Y, Sp1, and the retinoblastoma protein and repression by Sp3**. *Neoplasia* 2000, **2:**531-539.

6. Wu J, Metz C, Xu X, Abe R, Gibson AW, Edberg JC, Cooke J, Xie F, Cooper GS, Kimberly RP: **A Novel Polymorphic CAAT/Enhancer-Binding Protein {beta} Element in the FasL Gene Promoter Alters Fas Ligand Expression: A Candidate Background Gene in African American Systemic Lupus Erythematosus Patients**. *J Immunol* 2003, **170:**132-138.
